# Plastic Change in the Auditory Minimum Threshold Induced by Intercollicular Effects in Mice

**DOI:** 10.1155/2016/4195391

**Published:** 2016-01-17

**Authors:** Hui-Xian Mei, Jia Tang, Zi-Ying Fu, Liang Cheng, Qi-Cai Chen

**Affiliations:** ^1^School of Life Sciences and Hubei Key Lab of Genetic Regulation and Integrative Biology, Central China Normal University, Wuhan 430079, China; ^2^School of Sport, Hubei University, Wuhan 430062, China; ^3^Key Laboratory of Adolescent Cyberpsychology and Behavior, CCNU, Ministry of Education, Wuhan 430079, China; ^4^School of Psychology, Central China Normal University, Wuhan 430079, China

## Abstract

In the auditory pathway, the commissure of the inferior colliculus (IC) interconnects the two ICs on both sides of the dorsal midbrain. This interconnection could mediate an interaction between the two ICs during sound signal processing. The intercollicular effects evoked by focal electric stimulation for 30 min could inhibit or facilitate auditory responses and induce plastic changes in the response minimum threshold (MT) of IC neurons. Changes in MT are dependent on the best frequency (BF) and MT difference. The MT shift is larger in IC neurons with BF differences ≤2 kHz than in those with BF differences >2 kHz. Moreover, MTs that shift toward electrically stimulated IC neurons increase with the increasing MT difference between the two ICs. The shift in MT lasts for a certain period of time and then returns to previous levels within ~150 min. The collicular interactions are either reciprocal or unilateral under alternate stimulating and recording conditions in both ICs. Our results suggest that intercollicular effects may be involved in the acoustic experience-dependent plasticity of the MT of IC neurons.

## 1. Introduction

Auditory representation of the central auditory system in adult animals can be functionally reorganized when the acoustic environment is dramatically altered with relevant behaviors or through activation of the neuromodulation system [1–3]. Considerable evidence indicates that the inferior colliculus (IC), as a central auditory nucleus, can be continuously reshaped via an experience-dependent manner. The IC receives input from the auditory cortex (AC) through the descending auditory pathway [4, 5]. These corticofugal projections are believed to play an important role in the information processing and functional plasticity of the IC [6].

Corticofugal modulation studies on the IC show that the IC frequency map can be changed by repetitive acoustic stimulation, auditory conditioning, or focal cortical electric stimulation [7]. The best frequency (BF) shift in the IC usually increases when the acoustic stimulation is made behaviorally relevant by pairing with electrical stimulation [8]. This acoustic-electric stimulation also modulates the auditory sensitivity of IC neurons by changing their response minimum threshold (MT), dynamic range, best amplitude, best azimuth, and best duration. Through these modifications, the IC neurons are induced to shift toward the electrically stimulated AC neuron [9–12]. Cortical neurons have been suggested to mediate both a highly focused positive feedback to “matched” subcortical neurons while tuning to a particular acoustic parameter and a widespread lateral inhibition to “unmatched” subcortical neurons. This egocentric selection adjusts the response property of the IC depending on the auditory experience based on associative learning [8]. Moreover, collicular plasticity is augmented by basal forebrain and/or somatosensory cortical stimulation [13, 14].

One IC also receives inputs from the opposite IC; the commissural of IC (CoIC), which interconnects two ICs, mediates the intercollicular effects on sound information processing [15–18]. Our recent studies show that real-time focal electrical stimulation of one IC produces widespread inhibition and focused facilitation of the opposite IC in the amplitude domain [19–21]. In this study, we further explore the role of intercollicular effects via CoIC on the functional plasticity of the amplitude domain of IC neurons by auditory conditioning that acoustic stimulation paired by 30 min of IC focal electrical stimulation. Specifically, we study how the MTs of IC neurons change in an experience-dependent manner by intercollicular effects.

## 2. Materials and Methods

### 2.1. Animal Preparation and Surgery

A total of 68 two- to three-month-old adult mice (*Mus musculus* KM, supplied by the Center for Disease Control and Prevention of Hubei Province, China) was used for this study. Of these mice, 28 were females and 40 males, with body weights (BW) of 20–25 g. The experiments were conducted with the approval of the Institutional Animal Care and Use Committee of Central China Normal University, Wuhan, Hubei, China. The surgical procedures employed were basically identical to those described in previous studies [22, 23]. Briefly, the flat head of a 2.0 cm nail was glued onto the exposed skull of each Nembutal-anesthetized mouse (60–90 mg/kg b.w.) with acrylic glue and dental cement. The exposed tissue was treated with an antibiotic (Neosporin) to prevent inflammation. After 1-2 h, the animal was tied to a metal plate inside a custom-made, double-wall, sound-proof room (temperature: 28–30°C). The ceiling and walls of the room were covered with 2 cm polyurethane foam to reduce echoes.

After fixing the head with a set screw and orienting the eye-snout line to 0° in azimuth and 0° in elevation relative to the frontal auditory space, small holes (200–500 *μ*m) were bored into the skull above each IC for orthogonal insertion of custom-made tungsten electrodes (see below) and a 2 M NaCl glass pipette electrode (tip diameter: <1 *μ*m; impedance: 5–10 MΩ). These electrodes were applied for focal electrical stimulation and recording of sound-activated responses in the central nucleus of the IC, respectively. The depths of the recorded IC neurons were read from the scale of two microdrives (David-Kopf, Model 640, USA). A common indifferent electrode (silver wire) was placed at nearby temporal muscles. Additional doses of anesthetics (one-fourth of the original dosage) were administered during the later phases of recording when the animal showed signs of discomfort. A local anesthetic (lidocaine) was applied to the open wound area to reduce pain. Whenever possible, each animal was subjected to one to three recording sessions on separate days, and each recording session typically lasted for 2–6 h.

### 2.2. Stimulation and Isolation of Acoustically Evoked IC Neurons

For acoustic stimulation, continuous sine waves from a function generator (GFG-8016G, Good Will Inst Co., Ltd., Bayan Lepas, Penang, Malaysia) were formed into 40 ms pure tones (5 ms rise-decay times) with a custom-made tone burst generator (electronic switch) driven by a stimulator (Model SEN-7203, Nihon Kohden Co., Shinjuku, Tokyo, Japan) and delivered at 2 pulses per second. The tone pulses were then amplified (custom-made amplifier) after passing a decade attenuator (LAT-45, Leader, Kohokuku, Yokohama, Japan) before they were fed into a small loudspeaker (AKG Model CK 50, 1.5 cm in diameter, 1.2 g, and frequency response: 1–100 kHz). The loudspeaker was calibrated with a 1/4-inch microphone (4939, B&K, Denmark) placed at the mouse's ear using a measuring amplifier (2610, B&K, Denmark). The output of the loudspeaker was expressed in decibel sound pressure level (dB SPL) in reference to the 20 *μ*Pa root mean square. A frequency response curve of the loudspeaker was plotted to determine the maximal available sound amplitude at each frequency. The maximal stimulus amplitude ranged from 95 dB to 120 dB SPL between 10 and 80 kHz but declined sharply to 80 dB SPL at 100 kHz thereafter.

Two insulated tungsten electrodes (FHC Inc., Bowdoin, ME, USA) were glued together (tip: <10 *μ*m; intertip distance: ≤100 *μ*m) to form a pair of tungsten electrodes. These electrodes were used to record sound-activated IC responses and focal electrical stimulation in the IC recording site (4 ms train of four monophasic pulses of 0.1 ms with 0.9 pulse gaps at 2 train/s, 5–50 *μ*A) using a stimulator (Model SEN-7203, Nihon Kohden Co., Tokyo, Japan) and stimulus isolation unit (Model Nihon Kohden Co., Tokyo, Japan).

During the experiment, a 40 ms sound was delivered (at 2 p/s) from the loudspeaker placed 30 cm away from the animal and 60° contralateral to the recording site. An IC neuron was isolated (first IC neuron, designated as the ipsilateral IC neuron) with a pair of custom-made tungsten electrodes, and its BF and MT were audiovisually measured by systematically changing the frequency and amplitude of the sound pulses. The sound frequency that elicited the neuron's response at the lowest amplitude was defined as the BF. The threshold at the BF was defined as the MT. At the MT, the neuron, on average, responds with 50% probability to BF pulses.

The acoustically evoked responses of an IC neuron in the other IC (second IC neuron, designated as the contralateral IC neuron) was then isolated with a 2 M NaCl glass electrode after moving the loudspeaker 60° contralateral to the isolated IC neuron. After determining the contralateral IC neuron's BF and MT, the neuron's response to the BF sound pulses delivered at 10 dB above the MT was recorded as a control response. The neuron's response was then monitored again during focal electrical stimulation of the first isolated IC neuron through the custom-made tungsten electrodes. The focal electrical stimulation was delivered between 5 and 50 *μ*A and at a randomly chosen interstimulus interval (ISI). When the response of the contralateral IC neuron became affected during the focal electrical stimulation of the ipsilateral IC neuron, the electrical stimulation current was then fixed at 25 *μ*A and the ISI was adjusted systematically to determine the optimal ISI that produces the maximal modulation effect. At the optimal IPI, the intercollicular effect was then studied with focal electrical stimulation applied at 25 *μ*A and 10 trains/s, synchronized with the BF sound of the ipsilateral IC neuron delivered at 10 dB above the neuron's MT for 30 min. The rate-amplitude function (RAF) is measured through the neuron's number of impulses obtained after a BF sound was delivered at MT and 10 dB increments above the MT. For convenience of description, the electrically stimulated ipsilateral IC neuron is hereafter referred to as IC_ES_ neuron and the contralateral IC neuron, whose response was modulated, is hereafter referred to as IC_Mdu_ neuron.

To study the plasticity of the responses of the IC_Mdu_ neuron, we monitored 29 IC_Mdu_ neuron MTs and RAFs progressively at 0, 30, 60, 90, 120, and 150 min after 30 min of focal electrical stimulation of the IC_ES_ neuron.

Throughout the study course, 10 pairs of neurons in both ICs (i.e., 10 neurons in each IC) were isolated with custom-made tungsten electrodes such that each neuron could be electrically stimulated alternatively to study the reciprocal modulation of the acoustically evoked responses of each neuron. Focal electrical stimulation was applied in one IC neuron to determine the modulation effect on the responses of the other IC neuron. Then, the experimental procedures were switched such that the other IC neuron was electrically stimulated and the modulation effect on the response of the initially electrically stimulated IC neuron was monitored.

### 2.3. Data Collection and Analysis

Each IC neuron's response under different stimulation conditions was amplified (ISO-DAM, WPI, USA), band-pass filtered (Krohn-Hite 3500), and then fed through a window discriminator (WPI 121) before being sent to an oscilloscope (TDS210, Tek, USA) and an audiomonitor (Grass AM9, USA). The neuron's response data was also sent to a computer (Kaitian 4600, Lenovo, China) to generate peristimulus-time histograms (PSTHs) (bin width: 250 *μ*s; sampling period: 150 ms) for 32 sound presentations. The total number of impulses in each histogram was used to quantify the neuron's response under each stimulus condition.

All data obtained under different stimulation conditions were processed and plotted using Sigmaplot 2000. These data were then quantitatively examined and statistically compared using SPSS 13.0 (Student's *t*-test at *p* < 0.05).

## 3. Results

Among the responses of the 123 IC_Mdu_ neurons isolated, those of 88 neurons were modulated by 30 min IC_ES_ focal electrical stimulation. The ranges (mean ± standard deviation (SD)) of the BFs and MTs and recording depths of these IC_Mdu_ neurons were 8.4–35.2 (17.0 ± 5.7) kHz, 16–84 (55.6 ± 14.9) dB SPL, and 228–1928 (1062.3 ± 374.6) *μ*m, respectively.

**Figure 1:**
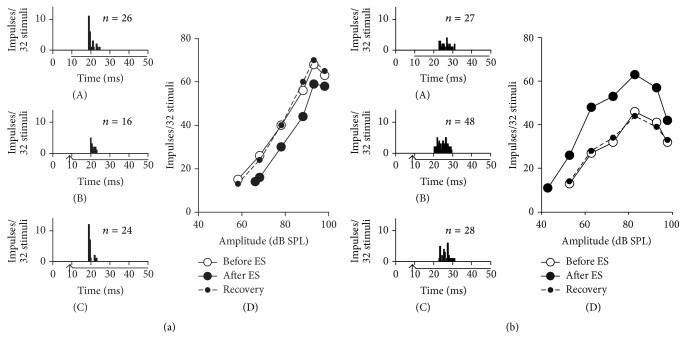
Inhibitory (a) and facilitatory (b) intercollicular effects on the responses of IC neurons. PSTHs showing the IC_Mdu_ neuron responses to BF sound stimulus (horizontal bar under abscissa) delivered at 10 dB above MT before ((a)(A), (b)(A)), after ((a)(B), (b)(B)), and during recovery ((a)(C), (b)(C)) from the 30 min IC_ES_ electrical stimulation (upward arrows under the abscissas). The RAFs of inhibited ((a)(D)) and facilitated ((b)(D)) IC_Mdu_ neurons obtained before (unfilled circle), after (filled circle), and during recovery (dashed lines) from 30 min of IC_ES_ electrical stimulation. *N*: number of impulses in each PSTH. The BF (kHz), MT (dB SPL), and recording depth (*μ*m) of the neurons were 16.5, 58.2, and 1200 for (a) and 12.2, 52.8, and 954 for (b).

IC_ES_ focal electrical stimulation produced a decrease in the number of impulses and an increase in the response latency of each of 63 (71.6%) inhibited IC_Mdu_ neurons (Figure 1(a)(A) versus Figure 1(a)(B)). The RAFs and MTs of these 63 neurons were suppressed and increased, respectively, by the focal electrical stimulation (Figure 1(a)(D)). Conversely, IC_ES_ focal electrical stimulation increased the impulse numbers and decreased the response latencies of 25 (28.4%) facilitated IC_Mdu_ neurons (Figure 1(b)(A) versus Figure 1(b)(B)). Moreover, the RAFs and MTs of these 25 neurons were facilitated and decreased, respectively (Figure 1(b)(D)). The inhibition and facilitation evoked by IC_ES_ focal electrical stimulation eventually deteriorated after a certain period (Figure 1(a)(A) versus Figure 1(a)(C); Figure 1(b)(A) versus Figure 1(b)(C)).

**Figure 2:**
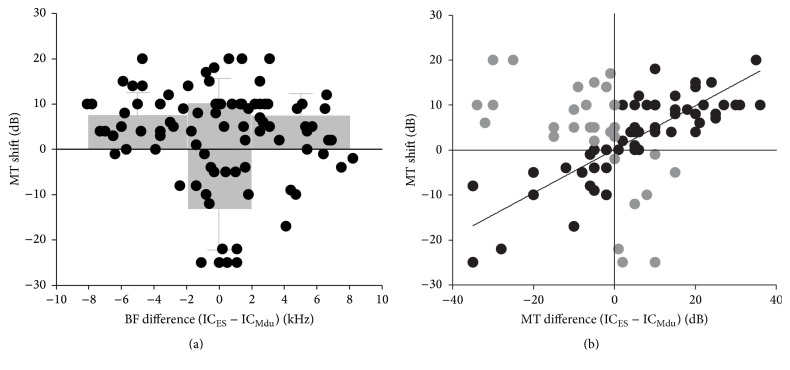
Scatter plots showing the MT shift that resulted from IC_ES_ electrical stimulation against BF (a) and the MT difference (b) between IC_ES_ and IC_Mdu_ neurons. The gray boxes and bars in (a) represent the mean ± SD of the MT shifts of these IC_Mdu_ neurons in the corresponding range of BF difference. The solid line in (b) is a regression line; the centripetal MT shifts (solid circles) evoked by IC_ES_ electrical stimulation were significantly related (*p* < 0.01), whereas the centrifugal MT shifts (gray circles) were unrelated (*p* > 0.05) to the MT difference.

Analysis of all 88 IC_Mdu_ neurons confirmed that the changes in the response MTs evoked by the IC_ES_ focal electrical stimulation of the IC_Mdu_ neurons were closely related to the BF and MT differences between the IC_ES_ and IC_Mdu_ neurons.  Figure 2(a) shows that, after electrical stimulation, the inhibited IC_Mdu_ neurons that produced an increased MT (shifted upward) hold BF differences (0–8 kHz) between the IC_ES_ and IC_Mdu_ neurons. However, most of the facilitated IC_Mdu_ neurons' MTs decreased (shifted downward) when the BF differences between the IC_ES_ and IC_Mdu_ neurons ≤2 kHz. On average, the mean MT changed by 11.3 ± 7.5 dB (increased by 10.3 ± 5.4 dB and decreased by 13.0 ± 9.2 dB) when the BF differences were ≤2 kHz. By contrast, when BF difference > 2 kHz, the mean MT changed by 7.5 ± 5.1 dB (IC_ES_ BF < IC_Mdu_ BF) or 7.4 ± 5.0 dB (IC_ES_ BF > IC_Mdu_ BF). These changes were both significantly smaller than those observed when the BF differences were ≤2 kHz (*t*-test, *p* < 0.05).

Figure 2(b) displays that IC_ES_ focal electrical stimulation induced the MT of the IC_Mdu_ neuron to shift toward (centripetal; first and third quadrants in Figure 2(b)) or away (centrifugal; second and fourth quadrants in Figure 2(b)) from the MT of IC_ES_ neuron. Linear regression analyses indicated that the MT shift increased with increasing MT difference between the IC_ES_ and IC_Mdu_ neurons only when the MT of the inhibited IC_Mdu_ neurons was smaller than that of the IC_ES_ neurons (*r* = 0.50, *p* < 0.01) or the MT of facilitated IC_Mdu_ neurons was larger than that of the IC_ES_ neurons (*r* = 0.63, *p* < 0.01). However, MT shifts did not correlate with MT differences when the MTs of the inhibited IC_Mdu_ neurons were larger than those of the IC_ES_ neurons (*r* = 0.28, *p* > 0.05) or the MTs of the facilitated IC_Mdu_ neurons were smaller than those of the IC_ES_ neurons (*r* = 0.15, *p* > 0.05). Overall, a significant correlation was noted between centripetal MT shift and MT difference but not between centrifugal MT shift and MT difference.

**Figure 3:**
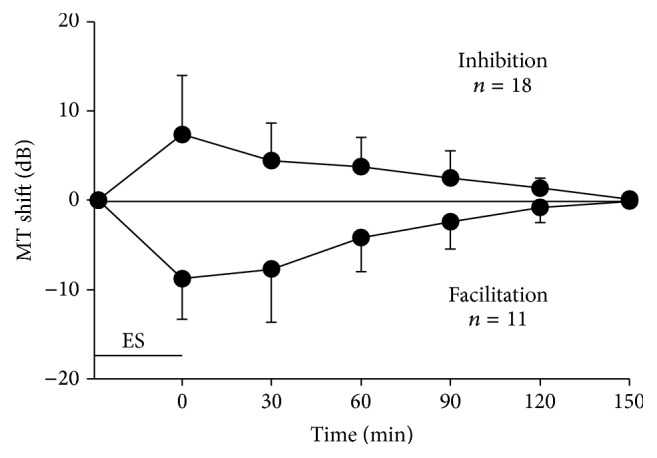
Time course of the variation in MT shift of the inhibited and facilitated IC_Mdu_ neurons after 30 min IC_ES_ focal electrical stimulation (indicated with short horizontal bar). *n* = number of IC_Mdu_ neurons; vertical bar = standard deviation.

To determine the time course of the modulation of the IC_Mdu_ neuron responses, we measured the MTs of 29 IC_Mdu_ neurons at different time frames after 30 min of IC_ES_ focal electrical stimulation. As shown in Figure 3, after IC_ES_ focal electrical stimulation, the increasing MT of the inhibited IC_Mdu_ neuron (*n* = 18) and decreasing MT of the facilitated IC_Mdu_ neuron (*n* = 11) both appeared to be the largest right after 30 min of IC_ES_ focal electrical stimulation (i.e., zero time of x-coordinate). The shifted MT then gradually returned to the control value (measured before IC_ES_ focal electrical stimulation) within ~150 minutes (Figure 3). Among the 29 IC_Mdu_ neurons studied, the recovery time of the MT shift induced by the 30 min IC_ES_ focal electrical stimulation was within 30 min in five neurons, 60 min in eight neurons, 90 min in eight neurons, 120 min in five neurons, and 150 min in three neurons.

**Figure 4:**
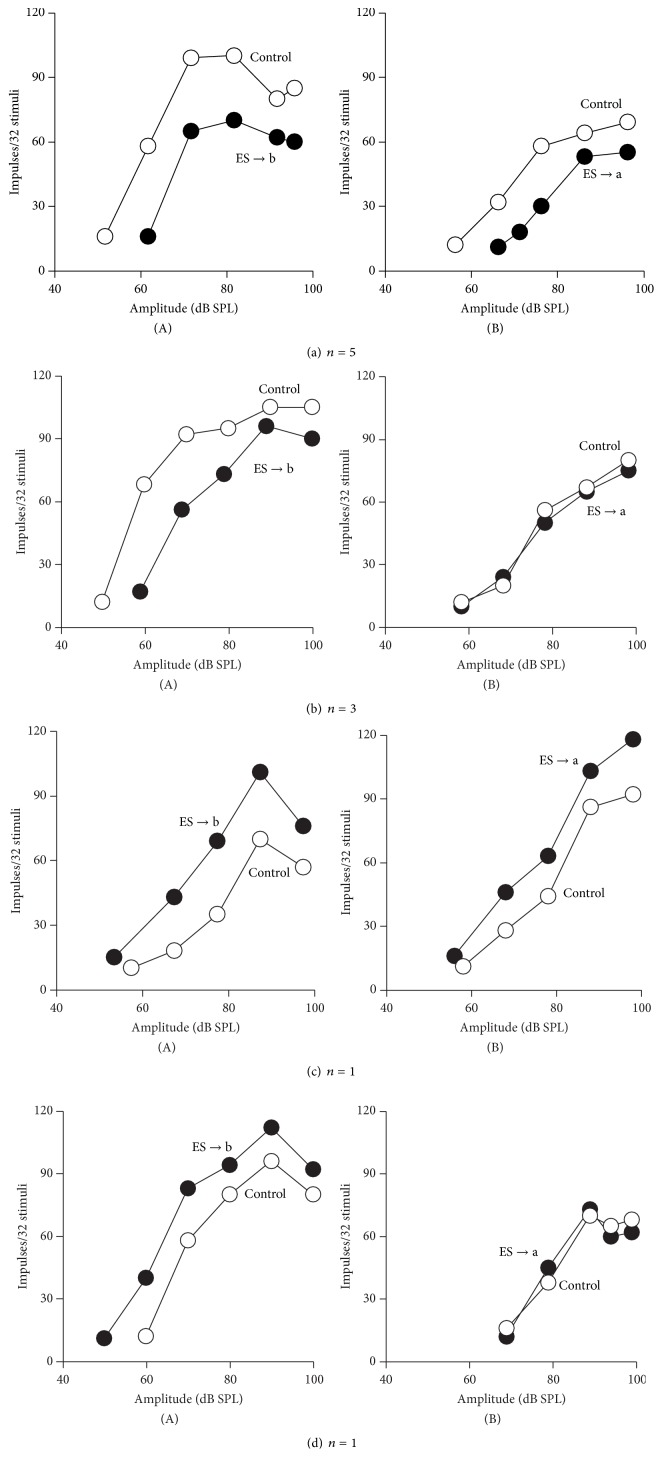
RAFs of the four pairs of IC neurons obtained before (unfilled circles) and after 30 min (filled circles) focal electric stimulation of each IC neuron. (a) The responses of both neurons (a)(A) and (a)(B) were reciprocally inhibited during the focal electrical stimulation of each IC neuron (ES → b and ES → a) and resulted in lowered RAFs (filled versus unfilled). (b) By contrast, reciprocal focal electric stimulation only lowered the RAF of neuron (b)(A) but not the RAF and MT of the other neuron (b)(B). (c) The responses of both neurons (c)(A) and (c)(B) were reciprocally facilitated during the focal electrical stimulation of each neuron (ES → b ES → a). This occurrence elevated the RAF (filled versus unfilled). (d) Reciprocal focal electric stimulation only elevated the RAF of one neuron (d)(A) but not the other neuron (d)(B). The respective BF (kHz), MT (dB SPL), and recording depth (*μ*m) of these eight IC neurons were 11.6, 52, and 969 for (a)(A) and 12.4, 56, and 1230 for (a)(B); 14.1, 50, and 1063 for (b)(A) and 8.4, 58, and 1509 for (b)(B); 9.8, 57, and 859 for (c)(A) and 11.7, 58, and 794 for (c)(B); and 9.8, 60, and 1321 for (d)(A) and 9.7, 69, and 1274 for (d)(B).

Given the time constraint and holding of the recorded IC neurons, we only studied the reciprocal intercollicular effects on the modulation of the RAFs and MTs of 10 pairs of IC neurons using alternative IC_ES_ focal electrical stimulation. As shown in Figure 4, the RAFs of four representative pairs of IC neurons were sequentially measured before (unfilled circles) and after (filled circles) the 30 min of focal electrical stimulation of each IC neuron. The responses of the five pairs of IC neurons were reciprocally inhibited during alternative focal electrical stimulation of each neuron (Figure 4(a); ES → b, ES → a), resulting in lower RAF and rising MT (filled versus unfilled). In another three pairs of IC neurons, alternative focal electrical stimulation only lowered the RAF and raised the MT of one neuron but not those of the other neurons (Figure 4(b); ES → b, ES → a). Similarly, the responses of a pair of IC neurons were reciprocally facilitated during alternative focal electrical stimulation, resulting in elevated RAFs and lowered MTs (Figure 4(c); ES → b, ES → a). However, in another pair of IC neurons, alternative focal electrical stimulation only elevated the RAF and lowered the MT of one neuron but not those of the other neuron (Figure 4(d); ES → b, ES → a).

## 4. Discussion

### 4.1. Intercollicular Effects Activated by IC_ES_ Focal Electrical Stimulation

This study demonstrated that inhibited IC_Mdu_ neurons exhibited decreased impulse numbers and increased response latencies and MTs after 30 min of IC_ES_ focal electrical stimulation. By contrast, facilitated IC_Mdu_ neurons showed the opposite response (Figure 1). The inhibitory and facilitatory CoIC has been proven to interconnect the two ICs on both sides of the dorsal midbrain [24–26]; hence, the above-mentioned study findings are likely due to the fact that IC_ES_ focal electrical stimulation weakens and strengthens the effectiveness of a given sound stimulus through inhibition and excitation of inhibited and facilitated IC_Mdu_ neurons, respectively. These inhibitory and facilitatory types of modulation activated by the 30 min IC_ES_ focal electrical stimulation are similar to those demonstrated in a previous work [20, 21]. In the mentioned real-time study, intercollicular effects were shown to be mediated through widespread inhibition and focused facilitation [20, 21]. In the current study, most of the facilitated IC_Mdu_ neurons displayed BF differences smaller than 2 kHz, whereas the inhibited IC_Mdu_ neurons showed a wide range of BF differences (0–8 kHz) (Figure 2(a)). The asymmetric distribution of facilitatory and inhibitory interactions is possibly determined by the specific CoIC projections between two ICs. The minority of electrically activated IC_ES_ neurons possibly send mono- or multisynaptic excitatory projections to the IC_Mdu_ neurons in corresponding frequency laminae. By contrast, the majority of the electrically activated IC_ES_ neurons possibly send multisynaptic inhibitory projections to the IC_Mdu_ neurons in wide frequency laminae. The widespread inhibition between two ICs is probably involved in sound localization in the azimuth by increasing thresholds of the contralateral IC neurons. Furthermore, the focused facilitation in corresponding frequency laminae between two ICs would benefit the behavioral binaural sound experience and discrimination of voice without distortion.

### 4.2. Plastic Change in MT Induced by Intercollicular Effects

We found that the MT shift induced by 30 min IC_ES_ focal electrical stimulation was dependent on the BF difference between IC_ES_ and IC_Mdu_ neurons. MT shifts with BF differences ≤2 kHz in the IC_Mdu_ neurons were larger than those with BF differences >2 kHz (Figure 2(a)). According to the topographical organization of the CoIC between the ICs, the commissural neurons in the central nucleus of IC send divergent projections to the equivalent frequency laminae in the central nucleus of the opposite IC [20]. Moreover, the density of this projection is greatest between the corresponding points [20]. Therefore, the intercollicular effects evoked by focal electrical stimulation should be stronger in the equivalent frequency laminae between two ICs. In addition, after 30 min of IC_ES_ focal electrical stimulation, intercollicular effects induced the MT shift of an IC_Mdu_ neuron toward or away from the MT of electrically stimulated IC_ES_ neuron. In particular, the centripetal MT shift increased with the increase in MT difference between the IC_ES_ and IC_Mdu_ neurons. By contrast, the centrifugal MT shift appeared to be arbitrary (Figure 2(b)). The results of the MT based on specific CoIC projections between two ICs were different from the egocentric selection of corticofugal modulation on IC in mouse. In the latter case, the involved IC neuron showed a nearly symmetric shift of MT toward the stimulated cortical neuron only when the BFs of the IC neuron and cortical neuron were very close [10]. We are uncertain if this discrepancy in observation is simply due to sampling bias or functions of the different auditory centers. In our previous study on the intercollicular effects on frequency domain, focal IC_ES_ electrical stimulation produced corticofugal-like modulation on the BF shift of IC_Mdu_ neurons [27]. Considering that tone is a quality of particular importance in discriminating sound signals in nature, we suggest that modulation of intercollicular effects at the subcortical level is more responsible for frequency than for MT, which represents the auditory sensitivity of neurons.

The MT shifts induced by intercollicular effects lasted for certain periods of time in our study. MT shift was greatest at the end of the 30 min IC_ES_ focal electrical stimulation and returned to the control condition within ~150 minutes (Figure 3). These findings are basically the same as plastic changes in the IC induced by the activation of corticofugal system. Therefore, the intercollicular effects may also contribute to acoustic experience-dependent plasticity in the IC under auditory conditioning achieved by acoustic stimulation paired by 30 min IC focal electrical stimulation. Moreover, the effects may superficially adjust the amplitude map of the IC by auditory experience based on associative learning and enhance the neural representation of MT in ICs in a colliculus-specific manner.

### 4.3. Reciprocal Modulation between Two ICs

After alternative focal electrical stimulation and recording, the intercollicular effects did not always produce reciprocal modulation on paired neurons in both ICs (Figures 4(a) and 4(c) versus Figures 4(b) and 4(d)). These observations indicate that intercollicular effects are either reciprocal or unilateral. However, a previous anatomic study suggested that the interconnections between the ICs through their commissure were complementary rather than reciprocal. This notion is suggested by a previous report in which, after horseradish peroxidase (HRP) was deposited in CoIC, regions of the IC supplying fibers to the commissure were found to not be the main targets of the fibers' terminals [28]. The IC received a large number of unilateral and bilateral ascending inputs from many lower auditory nuclei as well as the CoIC from the contralateral IC. Thus, these crossed or uncrossed inputs were processed in the IC and shaped the binaural property of the IC neuron [20, 29, 30]. We believe that reciprocal intercollicular effects could benefit significantly from integrating binaural information. This action contributes to better sound location and spatial auditory sensitivity of the IC neuron.

## References

[1] Blake D. T., Heiser M. A., Caywood M., Merzenich M. M. (2006). Experience-dependent adult cortical plasticity requires cognitive association between sensation and reward. *Neuron*.

[2] Thiel C. M. (2007). Pharmacological modulation of learning-induced plasticity in human auditory cortex. *Restorative Neurology and Neuroscience*.

[3] Fuchs E., Flügge G. (2014). Adult neuroplasticity: more than 40 years of research. *Neural Plasticity*.

[4] Saldaña E., Feliciano M., Mugnaini E. (1996). Distribution of descending projections from primary auditory neocortex to inferior colliculus mimics the topography of intracollicular projections. *Journal of Comparative Neurology*.

[5] Winer J. A. (2006). Decoding the auditory corticofugal systems. *Hearing Research*.

[6] Bajo V. M., King A. J. (2013). Cortical modulation of auditory processing in the midbrain. *Frontiers in Neural Circuits*.

[7] Suga N., Xiao Z., Ma X., Ji W. (2002). Plasticity and corticofugal modulation for hearing in adult animals. *Neuron*.

[8] Gao E., Suga N. (1998). Experience-dependent corticofugal adjustment of midbrain frequency map in bat auditory system. *Proceedings of the National Academy of Sciences of the United States of America*.

[9] Jen P. H.-S., Zhou X. M. (2003). Corticofugal modulation of amplitude domain processing in the midbrain of the big brown bat, *Eptesicus fuscus*. *Hearing Research*.

[10] Yan J., Ehret G. (2002). Corticofugal modulation of midbrain sound processing in the house mouse. *European Journal of Neuroscience*.

[11] Zhou X. M., Jen P. H. S. (2007). Multi-parametric corticofugal modulation of sub-cortical auditory selectivity in the midbrain of bats. *Journal of Neurophysiology*.

[12] Ma X., Suga N. (2001). Corticofugal modulation of duration-tuned neurons in the midbrain auditory nucleus in bats. *Proceedings of the National Academy of Sciences of the United States of America*.

[13] Ma X., Suga N. (2003). Augmentation of plasticity of the central auditory system by the basal forebrain and/or somatosensory cortex. *Journal of Neurophysiology*.

[14] Zhang Y., Hakes J. J., Bonfield S. P., Yan J. (2005). Corticofugal feedback for auditory midbrain plasticity elicited by tones and electrical stimulation of basal forebrain in mice. *European Journal of Neuroscience*.

[15] Saldaña E., Merchán M. A. (1992). Intrinsic and commissural connections of the rat inferior colliculus. *Journal of Comparative Neurology*.

[16] Malmierca M. S., Rees A., Le Beau F. E. N., Bjaalie J. G. (1995). Laminar organization of frequency-defined local axons within and between the inferior colliculi of the guinea pig. *Journal of Comparative Neurology*.

[17] Malmierca M. S., Hernández O., Falconi A., Lopez-Poveda E. A., Merchán M., Rees A. (2003). The commissure of the inferior colliculus shapes frequency response areas in rat: an in vivo study using reversible blockade with microinjection of kynurenic acid. *Experimental Brain Research*.

[18] Malmierca M. S., Hernández O., Rees A. (2005). Intercollicular commissural projections modulate neuronal responses in the inferior colliculus. *European Journal of Neuroscience*.

[19] Mei H.-X., Cheng L., Chen Q.-C. (2013). Neural interactions in unilateral colliculus and between bilateral colliculi modulate auditory signal processing. *Frontiers in Neural Circuits*.

[20] Mei H.-X., Cheng L., Tang J., Fu Z.-Y., Jen P. H.-S., Chen Q.-C. (2012). Modulation of amplitude sensitivity by bilateral collicular interaction among different frequency laminae. *Neuroscience Letters*.

[21] Mei H.-X., Cheng L., Tang J. (2012). Bilateral collicular interaction: modulation of auditory signal processing in amplitude domain. *PLoS ONE*.

[22] Tang J., Wu F.-J., Wang D., Jen P. H.-S., Chen Q.-C. (2007). The amplitude sensitivity of mouse inferior collicular neurons in the presence of weak noise. *Chinese Journal of Physiology*.

[23] Wang X., Jen P. H.-S., Wu F.-J., Chen Q.-C. (2007). Preceding weak noise sharpens the frequency tuning and elevates the response threshold of the mouse inferior collicular neurons through GABAergic inhibition. *Brain Research*.

[24] Moore D. R., Kotak V. C., Sanes D. H. (1998). Commissural and lemniscal synaptic input to the gerbil inferior colliculus. *Journal of Neurophysiology*.

[25] Marie R. L. S. (1996). Glutamatergic connections of the auditory midbrain: selective uptake and axonal transport of D-[^3^H]aspartate. *Journal of Comparative Neurology*.

[26] Smith P. H. (1992). Anatomy and physiology of multipolar cells in the rat inferior collicular cortex using the in vitro brain slice technique. *The Journal of Neuroscience*.

[27] Cheng L., Mei H.-X., Tang J., Fu Z.-Y., Jen P. H.-S., Chen Q.-C. (2013). Bilateral collicular interaction: modulation of auditory signal processing in frequency domain. *Neuroscience*.

[28] Aitkin L. M., Phillips S. C. (1984). The interconnections of the inferior colliculi through their commissure. *Journal of Comparative Neurology*.

[29] Kelly J. B., Li L. (1997). Two sources of inhibition affecting binaural evoked responses in the rat's inferior colliculus: the dorsal nucleus of the lateral lemniscus and the superior olivary complex. *Hearing Research*.

[30] Pollak G. D. (2012). Circuits for processing dynamic interaural intensity disparities in the inferior colliculus. *Hearing Research*.

